# The evolving landscape of pediatric obesity and metabolic dysfunction-associated steatotic liver disease

**DOI:** 10.3389/fped.2025.1675713

**Published:** 2025-10-28

**Authors:** Resthie R. Putri

**Affiliations:** ^1^ Department of Medical Epidemiology and Biostatistics, Karolinska Institutet, Stockholm, Sweden; ^2^ Department of Clinical Science, Intervention, and Technology, Karolinska Institutet, Stockholm, Sweden

**Keywords:** obesity, childhood obesity, MASLD, NAFLD, fatty liver, steatosis, pediatric, pediatric obesity

## Abstract

The global rise in pediatric obesity has paralleled an alarming increase in metabolic dysfunction-associated steatotic liver disease (MASLD), formerly known as non-alcoholic fatty liver disease (NAFLD). MASLD in children represents a significant public health concern due to its potential progression to advanced liver disease and its association with a myriad of cardiometabolic comorbidities. This review elucidates the risk and consequences of pediatric MASLD in the population of children with obesity. It also identifies critical research gaps and outlines future directions for the prevention, diagnosis, and treatment of pediatric MASLD.

## Introduction

Pediatric obesity is a serious and evolving public health problem as the rates continue to rise (1), the age of onset decreases (2), and higher degree of obesity has become more prevalent (3). The earlier onset and the greater obesity severity likely contribute to a growing number of children living with obesity-related comorbidities. By 2035, at least 36 million children are projected to have comorbidities attributable to obesity (1). This will dramatically affect pediatric and adult healthcare.

One of the most common comorbidities in pediatric obesity is metabolic dysfunction associated steatotic liver disease (MASLD), which previously was known as non-alcoholic fatty liver disease (NAFLD). Approximately half of the pediatric obesity population is estimated to have MASLD (4). Despite its high prevalence, pediatric MASLD is often undiagnosed (5, 6), and even patients with positive initial screening results frequently lack further evaluation (5).

Given the vast number of children living with obesity, identifying subgroups at the highest risk of developing MASLD is crucial. MASLD can be reversed if detected and managed in the early stage (7). Adult studies have indicated the interaction between sex and age modulates the risk of MASLD (8), yet such an interaction has not been explored in children. Several studies in children have attempted to identify factors associated with the risk of MASLD, including metabolic parameters (9–13) and perinatal factors (14, 15). However, the findings were inconclusive. Moreover, the cross-sectional design of most studies (9, 10, 12, 13) limits the inference of the disease course. Besides, results from single-center studies (9, 11, 12, 14) required cautious interpretation.

Beside risk stratification, understanding the long-term consequences of pediatric MASLD is essential to determine the clinical course of the disease and the care pathway. Yet, current knowledge of pediatric MASLD outcomes remains limited. In adult MASLD, while only a minority develop severe form of MASLD, these patients have high risk of liver-related mortality (16). Conversely, among adults with the milder form of MASLD, cardiometabolic disease is the leading cause of death (17). Whether the findings in adults can be extrapolated to pediatrics is uncertain. Children with MASLD have been suggested to have worse metabolic and liver outcomes than adults (18–22) given that they have early adiposity exposure and long time to develop complications. Yet, because previous studies in children were from selected populations of patients undergoing liver biopsy (18–21), had small sample size (21), or had only few individuals with the event outcomes (22), the precision of the estimates and generalizability are limited. The purpose of this review was to elucidate the risk and consequences of pediatric MASLD in the population of children with obesity.

### Epidemiology of pediatric obesity: growing in number in severity

Between 1990 and 2022 the global age-standardized prevalence of obesity in girls increased from 1.7% to 6.9%, while in boys it increased from 2.1% to 9.3% (23). In nearly 90% of countries worldwide, the prevalence of obesity among school-aged children and adolescents has doubled during this period (23). Moreover, obesity has also become more prevalent among preschool-aged children (24). The severity of obesity is also a public health concern. In the pediatric population, high degree of obesity (i.e., class II-III obesity) has increased 1.7-fold globally in the period 2007—2017 compared to 1967—2007 (3). In many European countries, 1 in 4 children with obesity had high degree of obesity (25).

### Definition of pediatric obesity: a chronic relapsing disease, not only a condition

The World Health Organization defines obesity as “a chronic complex disease defined by excessive fat deposits that can impair health” (26). Further, obesity has a relapsing nature in a similar sense as, for example, hypertension (27, 28). Hence, long-term care is necessary. Yet, whether obesity is a disease is not universally accepted. It has been argued that a high body mass index (BMI) without any signs or symptoms is not enough to establish obesity diagnosis. C*linical obesity* which is denoted as “a condition in which the risk to health associated with excess adiposity has already materialized and can be objectively documented by specific signs and symptoms reflecting biological alterations of tissues and organs, which are consistent with extant illness” was recently proposed (29). However, the *specific signs and symptoms* that define and measure this condition require further clarification. Also, it is uncertain if the term clinical obesity is appropriate for pediatric population considering that their symptoms are often subtle and cardiometabolic derangements often manifest in later childhood (30).

## Causes, risk factors, pathogenesis of obesity: not as simple as eating too much

**Figure 1:**
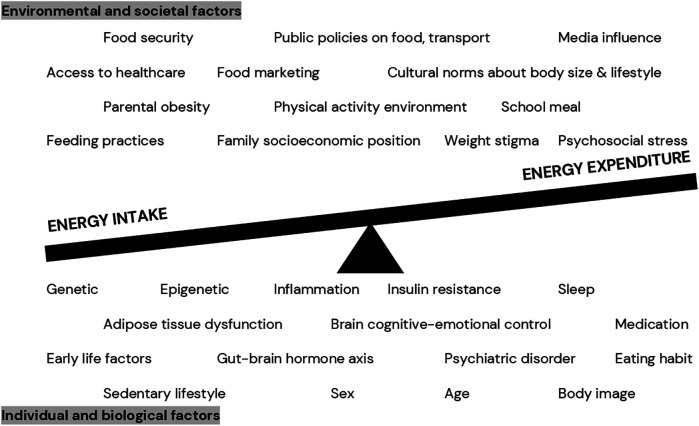
Complex relationship between various factors contributing to positive energy balance in pediatric obesity (31–34).

Obesity occurs as a long-term consequence of positive energy balance (i.e., when energy intake is more than energy expenditure). Nevertheless, the long-term positive energy balance is not caused by personal choice, but rather by a complex interaction between biological, individual, environmental, and societal factors (31–34), see Figure 1. In the molecular level, adipose tissue dysfunction, representing by adipocyte hypertrophy, adipose tissue expandability, hypoxia, and inflammation, does not only affect the development of obesity but also obesity-related comorbidities (35). Additionally, while causality remains unclear, gut dysbiosis has been shown to be associated with obesity development, both in animal models and human studies. For instance, Firmicutes/Bacteroidetes ratio seems to be higher in individuals with obesity than those with normal weight (36). Moreover, from environmental perspective, family socioeconomic position, local communities and culture, public policies on food and agriculture are indicated to influence the development of childhood obesity (31).

### Measurement and diagnosis of obesity: do we actually measure adiposity?

There is no perfect tool to measure excess adiposity in daily practice. Multicomponent models using densitometry are often considered the gold standard of fat mass measurement. However, they are unsuitable for pediatric population, impractical for routine practice, and not error-free (e.g., hydration status may bias the result) (37).

To date, BMI is still widely utilized as the primary indirect measure of fatness because it is simple, and it identifies correctly most children with excess adiposity (38). Yet, given some substantial limitations of BMI (e.g., affected by muscle mass and stature), the combination of medical history, physical examination, and anthropometric measurement is essential in pediatric obesity work-up (33).

Because children grow in height and weight, BMI in children should be compared with a growth reference adjusted for sex and age and can be measured in a standard deviation score (SDS). A comparison of some well-known growth references is presented in Table 1. Despite the differences in population source and obesity cut-off, these references have high agreement in classifying weight status (39). Overall, the International Obesity Task Force (IOTF) reference yields the lowest prevalence of obesity, while the WHO reference generates the highest (40). For epidemiological studies, the IOTF reference, which is based on pooled international data and linked to the adult obesity cut-off, is widely used (31, 41).

**Table 1:** Comparison of several growth references.

Reference	Population source	Age in years	Cut-off for overweight^a^	Cut-off for obesity
IOTF (41)	National survey from the UK, USA, the Netherlands, Brazil, Singapore, and Hong Kong.	2 to 18	1.31 SD in boys or 1.24 SD units in girls	2.29 SD in boys or 2.19 SD in girls
WHO (42)	Healthy breastfed children without any constraint to growth from Brazil, Ghana, India, Norway, Oman, and the USA.	0 to 5	2 SD	3 SD
WHO (43)	Children with healthy growth from national surveys in the USA.	5 to 19	1 SD	2 SD
CDC (44)	National health surveys from the USA.	2 to 20	1.04 SD ≅ 85th percentile	1.64 SD ≅ 95th percentile

CDC, center for disease control and prevention; IOTF, international obesity task force; SD, standard deviation; UK, United Kingdom; USA, United States of America; WHO, world health organization.

^a^ Overweight is also called as pre-obesity.

While BMI SDS is generally appropriate to define obesity in children, the use of BMI SDS to monitor weight status over time should be considered carefully, especially in children with high degree of obesity, because it may overestimate or underestimate improvement of weight status depending on the age of the children (45). Non-SDS BMI metrics (e.g., BMI percent of the 95th percentile) have been suggested as the preferrable anthropometric measure in monitoring treatment response (46).

### Comorbidities in pediatric obesity: almost all organs are affected

**Figure 2:**
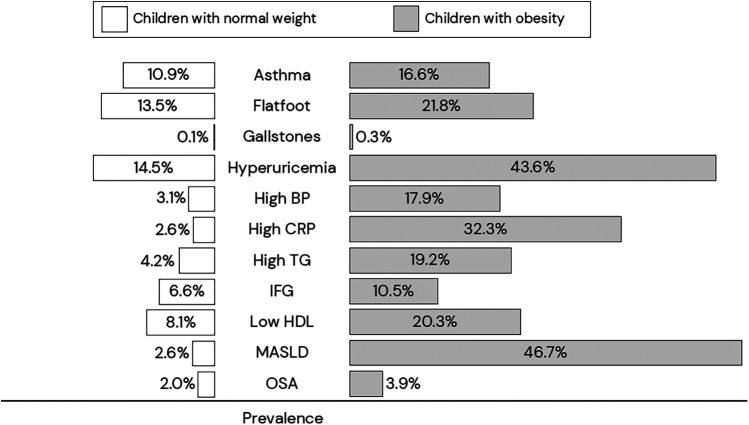
Prevalence of comorbidities in children with normal weight (white rectangle) vs children with obesity (gray rectangle) (47). BP, blood pressure; CRP, C-reactive protein; HDL, high-density lipoprotein cholesterol; IFG, impaired fasting hyperglycemia; MASLD, metabolic-associated steatotic liver disease; OSA, obstructive sleep apnea; TG, triglycerides.

Childhood obesity is associated with increased risk of various conditions or diseases in almost all organs (34). The pathophysiology of obesity-related comorbidities is complex, with low-grade chronic inflammation caused by excess adiposity playing an important role (34). Figure 2 shows some diseases or conditions more prevalent in children with obesity than in their peers with normal weight (47), with MASLD as one of the most common comorbidities in children with obesity.

### Pediatric obesity management: no shortcuts

Similar to other chronic diseases, childhood obesity requires comprehensive and long-term care (48–51). Moreover, weight regain or rebound is not uncommon (52). Nevertheless, the aim of obesity treatment is not only adiposity reduction and maintenance, but also includes early detection and management of comorbidities, and improvement of quality of life, self-image, and health behavior (33, 48, 49).

Until now, the primary treatment of childhood obesity has been behavioral intervention addressing dietary, physical activity, sleep, and sedentary behavior (31, 48, 49, 51). A reduction of at least 0.25 BMI SDS units during lifestyle intervention has been shown to improve cardiometabolic markers (53, 54). However, obtaining such a BMI SDS reduction is rather challenging. In Sweden, the average BMI SDS reduction after a year of treatment is −0.14 units, with approximately 30% of the patients obtaining a reduction of at least 0.25 SDS units within the first year of obesity treatment (55). A meta-analysis has also shown that the effect of behavioral intervention given in 6–12 months for school-aged children with obesity is generally modest (56) and indicated that longer continued care is required. Additionally, BMI SDS has methodological limitations, especially when utilized in subgroups with high degree of obesity (e.g., in adolescents with class III obesity, a great difference in BMI corresponds to a small difference in SDS) (45).

Other treatment options for pediatric obesity include anti-obesity medications and bariatric surgery. Liraglutide and Semaglutide, belonging to the group of glucagon-like peptide-1 (GLP-1) receptor agonists, have been approved in Europe as pharmacotherapy in adolescents from age 12 years (57, 58). Liraglutide, administered subcutaneously once daily, in combination with lifestyle interventions, demonstrates a mean reduction in BMI SDS of 0.23 units after 56 weeks of treatment among adolescents with obesity and previous poor response to lifestyle alone (59). Semaglutide, administered subcutaneously once weekly, in combination with lifestyle intervention, results in a mean reduction in BMI SDS of 1.1 units after 68 weeks of treatment among adolescents with obesity and overweight who had associated morbidity (60). Setmelanotide, a melanocortin-4 receptor agonist, has been approved for children with obesity due to rare genetic variants that disrupt the melanocortin pathway (61). Additionally, in adult obesity treatment, Tirzepatide, a glucose-dependent insulinotropic polypeptide and GLP-1 receptor agonist, has shown good efficacy. Its efficacy and safety in pediatrics are still investigated in an ongoing phase-3 trial (62). Other than medications, bariatric surgery has also been introduced as a treatment option for adolescents with severe obesity (63). Among 81 adolescents with severe obesity treated in the specialized pediatric treatment centers in Sweden, the mean BMI reduction was 13.1 kg/m^2^ over 5 years (64).

### A silent epidemic of MASLD

The estimated prevalence of pediatric MASLD varies depending on the diagnostic tools and the study population. A meta-analysis estimated an overall global prevalence of pediatric MASLD of 7.4% (95% CI: 4.2%–12.8%) in the general population and 52.5% (95% CI: 46.2%–58.7%) in the pediatric obesity population. Despite its high estimated prevalence, MASLD is often underscreened and underdiagnosed in the pediatric obesity population (5).

Among children with MASLD, the prevalence of MASH and fibrosis is challenging to establish given that liver biopsy is usually required to confirm MASH and fibrosis. Among children with biopsy-proven MASLD treated in tertiary care, approximately 20%–50% of them had MASH and 10%–20% had advanced fibrosis at the time of diagnosis (65). However, given the selected population, the prevalence is likely to overestimate the real-world prevalence.

### Definition of MASLD: one name with a large spectrum of disease phenotypes

According to a recent international consensus, MASLD is defined as “the presence of hepatic steatosis in conjunction with one cardiometabolic risk factor and no other discernible cause” (66). Despite being a single disease, MASLD encompasses a broad spectrum of phenotypes differentiated by liver histology (7, 66, 67), see Table 2. Worsening histology appears to be associated with higher mortality (68).

**Table 2:** Spectrum of pediatric MASLD adapted from NASPHAN (7) and ESPGHAN (67).

Phenotypes	Definition
Liver steatosis	Fat infiltration in the liver (at least 5% of hepatocytes) without evidence of significant inflammation or hepatocellular injury, with or without fibrosis.
MASH	Liver steatosis with inflammation, with or without fibrosis.
Cirrhosis	The advanced stage of fibrosis occurred in the setting of MASLD.

Not all patients with MASLD progress to cirrhosis. Adult studies indicated that only 1%–2% of patients with simple steatosis developed cirrhosis over 15–20 years of follow-up (69), whereas the corresponding proportion in children remains unknown. A study of Dutch children with high degree of obesity demonstrated that one of 24 patients who had steatosis at baseline developed advanced fibrosis after 7–13 years of follow-up (70). However, the relatively small number of individuals with MASLD in the study limits the precision of the findings.

### NAFLD, MAFLD, MASLD: are they interchangeable?

The term for steatotic liver disease in children has changed over time. After the first case report in the 1980s (71), the disease was called non-alcoholic fatty liver disease (NAFLD). This term has been long criticized for its ambiguity (i.e., “non-alcoholic” does not explain the etiology) and inaccuracy (i.e., alcohol disorder is not a major concern in children). In 2021, a new term called metabolic dysfunction associated fatty liver disease (MAFLD) and a change in diagnostic criteria was proposed (72). As the word “fatty” is considered stigmatizing, among other considerations, in 2023 a new term called metabolic dysfunction associated steatotic liver disease (MASLD) and its detailed diagnosis criteria for adults and pediatrics was established (66). Later in 2024, pediatric societies supported the new term MASLD yet highlighted some essential considerations in diagnosis that are unique in children (73).

NAFLD, MAFLD, and MASLD are not only about changes in nomenclature but also changes in the definition and diagnostic criteria. A key difference is that, unlike NAFLD, both MAFLD and MASLD require evidence of metabolic dysfunction. Table 3 shows the comparison of definition and diagnostic criteria for NAFLD, MAFLD, and MASLD.

**Table 3:** Comparison of definition and diagnostic criteria for NAFLD, MAFLD, and MASLD.

Definition, diagnosis, and etiologies	NAFLD (7, 67)	MAFLD (72)	MASLD (66, 73)
Definition	Chronic hepatic steatosis, in the absence of genetic/metabolic disorders, infections, steatogenic medications, ethanol consumption, or malnutrition.	Hepatic steatosis associated with metabolic dysfunction.	Hepatic steatosis in conjunction with one cardiometabolic risk factor and no other discernible cause.
Work-up or diagnostic criteria	Fatty liver disease indicated by ALT (7) or a combination of ALT and ultrasound (67), or liver biopsy, **AND** Test for exclusion of other main causes of liver steatosis (e.g., serum lactate, iron, ferritin, pyruvate, copper, ceruloplasmin, alpha 1-antitrypsin level, viral hepatitis panel, liver autoantibodies).	Hepatic steatosis detected either by imaging (with ultrasound or controlled attenuated parameter), blood biomarkers (ALT), or liver histology, **AND** • Excess adiposity based on BMI SDS or waist circumference percentile, *OR* • Prediabetes or type 2 diabetes, *OR* • At least two metabolic risk abnormalities based on blood pressure, plasma triglycerides, plasma HDL cholesterol, and triglycerides-to-HDL-ratio	Hepatic steatosis identified by ALT^a^, imaging or biopsy, **AND** At least 1 of 5 cardiometabolic criteria based on: • BMI SDS or waist circumference percentile • Serum glucose, HbA1c, already diagnosed or treated type 2 diabetes • Blood pressure or antihypertensive drug treatment • Plasma triglycerides or lipid-lowering treatment • Plasma HDL cholesterol or lipid-lowering treatment Children should also undergo investigation for autoimmune hepatitis, Wilson disease, viral hepatitis, alpha-1-antitrypsin deficiency, celiac disease, alcohol use in adolescents, and potential drug-induced liver injury^a^.
Mixed etiologies	Not emphasized.	MAFLD can stand alone or coexist with other liver disease.	The possibility of dual pathology (MASLD and other etiology) must always be considered in the investigation.

ALT, alanine aminotransferases; ESPGHAN, the European Society for Pediatric Gastroenterology Hepatology and Nutrition; MAFLD, metabolic dysfunction associated fatty liver disease; MASLD, metabolic dysfunction associated steatotic liver disease; NAFLD, non-alcoholic fatty liver disease; NASPGHAN, the North American Society for Pediatric Gastroenterology, Hepatology & Nutrition.

^a^ Additional diagnostic criteria highlighted by pediatric societies (26).

An important question is whether the previous findings on NAFLD or MAFLD studies can be extrapolated to MASLD. The short answer is yes, especially in obesity population. In general pediatric population in the United States, 80% of patients with NAFLD met the criteria for MAFLD, whereas all children with NAFLD fulfilled the MAFLD criteria in a Chinese pediatric obesity population (74). In a Taiwanese cohort of school-aged children, the prevalence of NAFLD and MASLD was equal within the study population (75). In general adult population in Sweden, 99.5% of patients with NAFLD met the MASLD criteria (76).

### Causes, risk factors, pathogenesis of MASLD: multiorgan crosstalk

Although not clearly understood yet, the most widely accepted pathogenesis of pediatric MASLD is called the “multiple-hit theory”, whereby MASLD occurs due to crosstalk between multiple organs, including the liver, adipose tissue, pancreas, and gut (77). Briefly, the ‘first hit’ is marked by free fatty acid accumulation in the liver (77). Sources of free fatty acids are dietary intake, lipolysis in adipocytes, and hepatic lipogenesis, which are associated with obesity and insulin resistance (78). The “second hit” is marked by inflammation and cell death due to lipotoxicity. The mechanism of how steatosis environment triggers inflammation remains unclear, but oxidative stress, mitochondrial dysfunction, proinflammatory cytokines imbalance, and dysbiosis of gut microbiota play roles (77, 79). The ‘third hit’ is the sequences of wound healing after inflammation and cell death. Hepatic stellate cells are activated, differentiate to myofibroblast, and subsequently, promote the regeneration of hepatocytes. However, when liver injury occurs repetitively, the regeneration process is impaired, leading to liver fibrosis (78).

Obesity is known as the greatest risk factor for MASLD (73, 80). The interplay between obesity and insulin resistance plays a major role in liver fat accumulation (77). Excessive fructose intake has also been suggested to promote liver steatosis and inflammation by inducing hepatic lipogenesis, increasing hepatic insulin resistance, and affecting the gut-liver axis (81). A large meta-analysis showed that added fructose intake from various food sources such as biscuits, cake, or sugar-sweetened beverages was associated increased risk of MASLD (82). Additionally, while findings on the association between different types of fat intake and MASLD were inconclusive (83), low-fat diet has been reported to be associated with regression of MASLD (84). This suggests the role of fat intake in MASLD pathogenesis. Moreover, the effect of maternal adiposity during pregnancy on the increased liver fat content in the offspring has also been indicated (85).

There are also some non-modifiable factors associated with pediatric MASLD, such as genetic variants, birth weight, and sex. Among all studied genetic variants, the patatin-like phospholipase domain containing 3 (PNPLA3) gene is the most established variant associated with an increased likelihood of liver steatosis and liver injury in children and adolescents (86). Birth weight (both low and high birth weight) and its association with MASLD occurrence and MASLD severity have also been reported (15, 87). However, whether the association between birth weight and MASLD is mediated by childhood obesity is unclear. In addition, boys have a higher likelihood of MASLD compared to girls (12, 13).

### In the absence of a good simple test, how to establish MASLD?

Until now, the gold standard to define the presence and severity of pediatric MASLD is liver biopsy. However, biopsy should not be performed in all children with suspected MASLD considering its invasiveness and that general anesthesia is commonly required in children (88, 89). Liver biopsy is recommended in particular cases, for instance to exclude other liver diseases (that cannot be excluded using non-invasive tests), to ascertain advanced disease or possibility of multiple liver diagnoses, before surgical treatment or potentially hepatotoxic medications, and in clinical trials (88, 89). Biopsy also has some drawbacks, such as sampling error (7) and relatively poor inter-rater reliability among hepatopathologists in assessing steatohepatitis (90).

Alanine aminotransferase (ALT), an enzyme found predominantly in hepatocytes, has been recommended by pediatric hepatology and obesity guidelines as a biomarker for MASLD screening (7, 48, 67, 73, 89, 91, 92). Despite its limitations (e.g., within-individual variability, mediocre sensitivity, not a direct marker of steatosis), ALT remains the primary biomarker for pediatric MASLD due to its availability, practicality, low cost, and accuracy compared to other existing non-invasive tests. Moreover, elevated ALT in children with excess adiposity is very likely due to MASLD (7, 73, 93). Nevertheless, ALT threshold for positive MASLD screening is various [e.g., a threshold of 22 U/L for girls and 25 U/L for boys was recommended by the Endocrine Society (91), 35 U/L by ESPGHAN (67), 44 U/L for girls and 52 U/L for boys by NASPGHAN and AAP (7, 48), 48 U/L by the Swedish pediatric guidelines (49)] or not mentioned (94). Also, guidelines’ recommendations vary in what to do when positive screening is found.

Unlike in adult MASLD, the use of prediction scores and imaging to detect steatosis or fibrosis is not as established in children. Some existing biomarkers or prediction scores to detect adult steatosis (fatty liver index score, NAFLD liver fat score) or fibrosis (e.g., aspartate aminotransferase-to-platelet ratio, Fibrosis-4 Index) have low accuracy or need further validations in children (92, 95). To detect steatosis in children, ultrasound has low accuracy in detecting mild steatosis (i.e., liver steatosis <30%) and longitudinal changes of steatosis (7, 48, 92). Better accuracy in quantifying steatosis in children is shown by controlled attenuation parameter (CAP) and magnetic resonance imaging-proton density fat fraction (MRI-PDFF) (96, 97). Yet, further studies to determine the CAP threshold are warranted, and MRI utilization is limited given its high cost and sedation-required for young children. To detect fibrosis, the performance of currently available imaging (e.g., transient elastography and magnetic resonance elastography) in pediatric populations needs to be investigated further (98). Non-invasive test with good accuracy in detecting MASLD in children is urgently needed.

**Figure 3:**
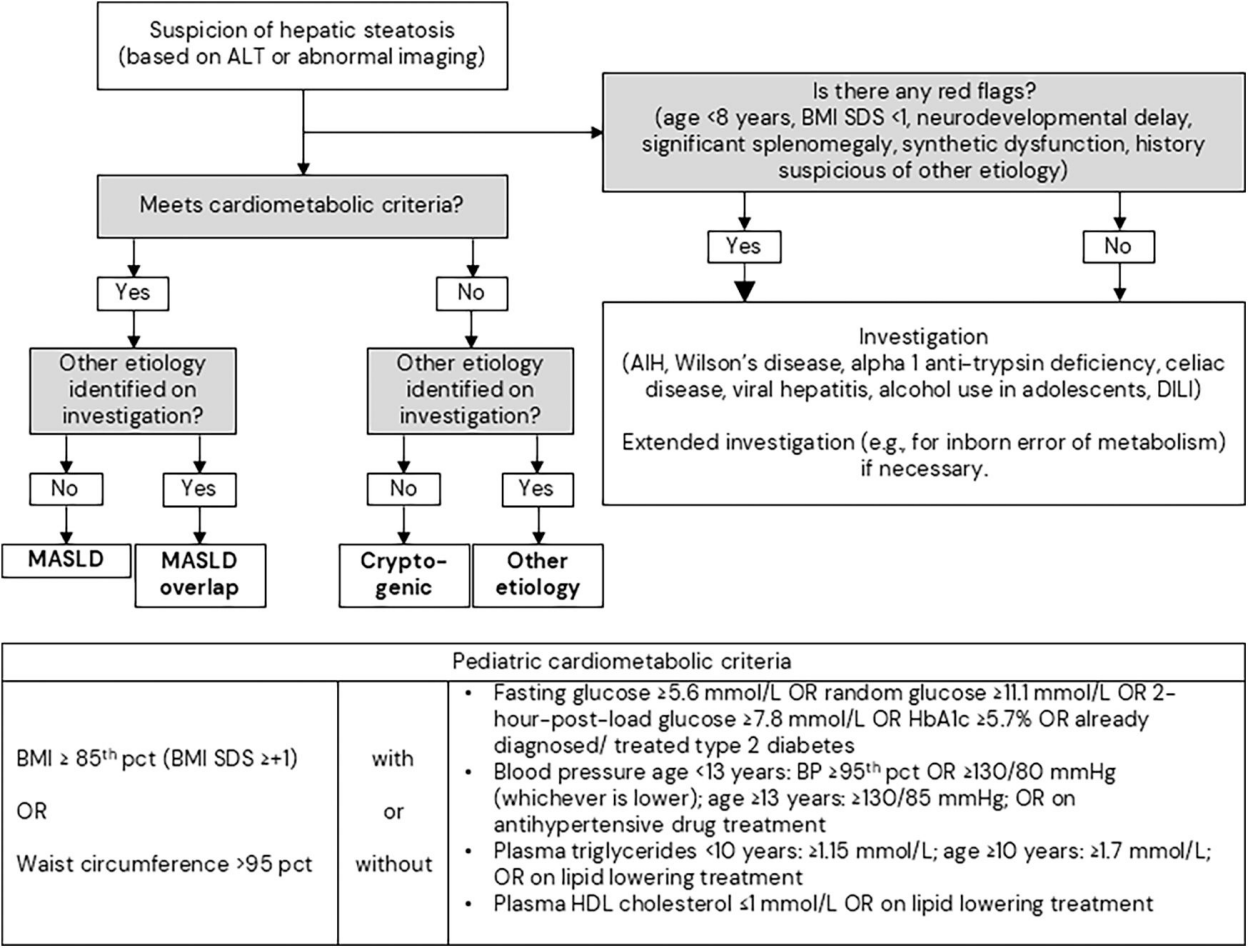
Diagnostic pathway for steatotic liver disease in children. The bigger arrow represents that immediate investigation is required. The figure is modified from the *European Society for Pediatric Gastroenterology, Hepatology and Nutrition (ESPGHAN); European Association for the Study of the Liver (EASL); North American Society for Pediatric Gastroenterology, Hepatology, and Nutrition (NASPGHAN); Pediatric steatotic liver disease has unique characteristics: A multisociety statement endorsing the new nomenclature. J Pediatr Gastroenterol Nutr. 2024* (73), used under a Creative Commons Attribution 4.0 International License. AIH, autoimmune hepatitis; ALT, alanine aminotransferases; BMI, body mass index; DILI, drug-induced liver injury; MASLD, metabolic dysfunction associated steatotic liver disease; pct, percentile; SDS, standard deviation score.

A diagnostic pathway for MASLD has recently been clarified by pediatric societies (Figure 3). In the diagnostic pathway (Figure 3), it is important to note that other causes of liver steatosis in children, including Wilson disease, autoimmune hepatitis, viral hepatitis, alpha 1-antitypsin deficiency, lysosomal acid lipase deficiency, should not be overlooked (99–101).

### Comorbidities of MASLD: liver and beyond

A vast literature on adult MASLD showed that MASLD is not only associated with the incidence of cirrhosis and hepatocellular carcinoma (102) but also associated with increased risk of other diseases, including type 2 diabetes (103), cardiovascular disease (104), chronic kidney disease (105), hypertension (106). Whether such comorbidities also occur in pediatric MASLD to a similar extent is uncertain.

With regards to end-stage liver disease, pediatric MASLD has been assumed to be more harmful than the adult type as the disease course starts much earlier (107). However, previous findings were conflicting (21, 70) and large longitudinal studies are lacking.

The association between pediatric MASLD and type 2 diabetes has been shown in a population of 892 children with biopsy-proven MASLD (108) and a national population of Israeli adolescents before military service (22). However, the selected population in the studies (22, 108) limits the generalizability and the small number of patients developing type 2 diabetes (22) makes the estimates uncertain. Limited data an association between MASLD in youth and conditions such as atherosclerosis, decreased bone mineral density, chronic kidney disease, and obstructive sleep apnea (109). Population-based longitudinal studies are required to confirm the findings.

### MASLD management: hits two targets with one arrow

Not only for obesity, lifestyle intervention also remains the cornerstone for pediatric MASLD management (7, 94). Reduction in BMI SDS through lifestyle intervention is associated with reduction of liver fat and transaminases (110, 111). In adults, Resmetirom, a thyroid hormone receptor beta-selective agonist, has shown good efficacy in MASH resolution and fibrosis improvement (112). Additionally, Semaglutide and Liraglutide seem to have promising effects on adult MASH resolution (113, 114). However, to date, there is no approved pharmacotherapy for pediatric MASLD. For adolescents with severe obesity and non-cirrhotic MASH, gastric bypass surgery followed by long-term follow-up can be considered (63).

### MASLD in pediatric obesity: risk factors and consequences

Male and older age are independently associated with increased risk for MASLD (12, 13). Furthermore, interaction between age and sex on the risk of MASLD was indicated (115); the risk seems to be increasing in boys with increasing age, while the risk tends to be constant in girls. While birthweight is known to be associated with MASLD in general pediatric population (15), a large multinational cohort of children showed that small for gestational age was associated with increased risk of MASLD and other cardiometabolic factors in children with obesity (116).

It has been suggested that early exposure to adiposity and MASLD may lead to worse liver outcomes (21, 107). A recent Swedish nationwide study found that children with obesity had an increased risk for major adverse liver outcomes compared to their peers in the general population (117). Moreover, as adult MASLD is known to be positively associated with type 2 diabetes (103, 118), we also showed that pediatric MASLD increases the risk and accelerates the onset of type 2 diabetes in children with obesity.

While relative weight loss in pediatric obesity has indeed beneficial effects, weight loss in children is complex given their weight and height are naturally growing over time. Several pediatric trials have shown that relative weight loss improves metabolic biomarkers (119) and that reduction of at least 0.25 BMI SDS units is clinically beneficial (53, 54). This beneficial effect of obesity treatment was confirmed in a real-world study showing that reduction of at least 0.25 BMI SDS units in a long-term pediatric obesity treatment reduced the risk of MASLD (120).

## Discussion

This review highlights the rapid evolution in our understanding of pediatric obesity and MASLD, from its escalating prevalence to the intricate molecular pathways that drive its progression. The reclassification of NAFLD to MASLD epitomizes a fundamental shift in scientific thought, emphasizing the metabolic underpinnings of the disease and setting the stage for more targeted research and clinical approaches.

When resources in management of pediatric obesity and MASLD are limited, risk-stratified care can be useful. MASLD screening in children with obesity should be prioritized in children with higher degree of obesity, impaired fasting glycemia, or elevated triglycerides. Moreover, perinatal factors, especially birth weight for gestational age, are important to assess in managing pediatric obesity because it is associated with increased risk of developing cardiometabolic diseases. MASLD in pediatric obesity should not be overlooked because it increases the risk of youth-onset type 2 diabetes considerably. Moreover, MASLD in pediatric obesity may contribute to an increased risk of major adverse liver outcomes.

The close link between degree of obesity, cardiometabolic derangement, pediatric MASLD, future risk of youth-onset type 2 diabetes, and severe liver disease underscores the need for collaborative multidisciplinary care. Current screening and treatment guidelines for pediatric MASLD vary widely and are rather inconsistent, contributing to many MASLD cases being undiagnosed or neglected.

In conclusion, pediatric MASLD represents a critical and growing health challenge with far-reaching consequences. While significant progress has been made in understanding its epidemiology and pathogenesis, substantial research gaps persist, particularly regarding long-term outcomes and effective, targeted therapies. Future research direction must prioritize finding accurate non-invasive tests to diagnose and monitor pediatric MASLD. Without reliable non-invasive tests, studies in pediatric MASLD would consistently be hampered by misclassification or selection bias, weakening the internal validity. Developing such a reliable non-invasive test would remarkably accelerate our understanding of pediatric MASLD. Furthermore, a unified international guideline from pediatric hepatology, endocrinology, and obesity experts is of importance to improve the holistic and comprehensive care for children with MASLD. Raising pediatric MASLD awareness and knowledge among healthcare providers (both liver and non-liver specialists) and the population at the greatest risk is important to halt disease progression.
